# Blessed Charity

**Published:** 1858-03

**Authors:** 


### BLESSED CHARITY.

A LADY subscriber, from the sunny South, sent us some money, a few days ago, as her "mite towards helping some poor person." That same night, cold and drearily as the wind whistled by the lattice, we appropriated it to the use of a poor widow. She was an American, had been left destitute, with two small children, girls. When in good health, and with plenty of work for tailors, she was barely able to meet her absolute necessities. But she was a martyr to rheumatism. These attacks threw her back, and it would be long weary weeks before she could make up the double loss, of not earning anything, and using what she had saved. Just at this time, her fingers were swollen and her feet so disabled, that she could neither work nor walk. All the family supplies were exhausted, and her monthly rent of three dollars was almost due, with the alternative of being turned out, unless paid in advance. "How much have you saved towards the month's rent?" "*Thirty-seven cents.*" "How will you make up the remainder?" "*Indeed, I cannot tell. My poor children! what will become of them?*"

When told that money was sent for her use, which, with her own efforts, would enable her to pay her rent for several months, the reader may imagine what a grateful joy lighted up her sad and careworn face, and what a mine of happiness was sprung by the "mite" of the "Southern widow for her Northern sister—a happiness sweeter than the veriest miser experiences when he adds another thousand to his hoard of gold. To live in comfort and in abundance in our own homes, should be the source of constant and heartfelt thanksgiving; but to have the means and the heart to happify the less fortunate of our kind, merits a higher and a purer gratitude.

---

*London Lancet*, STRINGER & TOWNSEND, New York, five dollars a year, is the cheapest monthly medical publication in the world, and with *Braithewaite's Retrospect*, and *Ranking's Abstract*, semi-annuals, at two dollars a year, should be taken by every educated physician, whose motto is "Progress."

*Sargent's School Monthly*, Boston, $1 a year, we heartily commend to every household. *Blackwood's Magazine* has an article of great interest on *Hunger and Thirst*. Col. R. J. Page, of South Carolina, has communicated to the *Farmer and Planter* on "Plantation Hygiene," embodying facts in relation to the connection between clearing lands and health, which should be published in every newspaper, showing why localities suddenly become unhealthful which had been remarkable for their salubrity. *The Home*, monthly, Buffalo, is well worthy of family patronage; every article in the last number is a good one. DIXON'S *Scalpel*, $1 a year, is the best quarterly on "Health" in the world. It is full of valuable practical, scientific information, and withal, so tart, true, and humorous, that the very reading of it is healthful.

*Westminster Review*, with the *London*, *Edinburgh*, *North British*, and *Blackwood*, are furnished at ten dollars a year, by LEONARD SCOTT & Co., 79 Fulton street, New York, affording more valuable periodical reading than can be obtained for an equal amount of money in any country. *Westminster* is infidel.

